# Pyrrolidine Dithiocarbamate Suppresses *Cutibacterium acnes*-Induced Skin Inflammation

**DOI:** 10.3390/ijms24054444

**Published:** 2023-02-23

**Authors:** Jin Hak Shin, Seon Sook Kim, Su Ryeon Seo

**Affiliations:** 1 Department of Molecular Bioscience, College of Biomedical Science, Kangwon National University, Chuncheon 24341, Republic of Korea; 2 Institute of Life Science, Kangwon National University, Chuncheon 24341, Republic of Korea; 3 Institute of Bioscience & Biotechnology, Kangwon National University, Chuncheon 24341, Republic of Korea

**Keywords:** *Cutibacterium acnes*, PDTC, skin inflammation, inflammasome, bone marrow-derived macrophages, IL-1β

## Abstract

*Cutibacterium acnes* (*C. acnes*), a Gram-positive anaerobic bacterium, proliferates in hair follicles and pores and causes inflammation in the skin of young people. The rapid growth of *C. acnes* triggers macrophages to secrete proinflammatory cytokines. Pyrrolidine dithiocarbamate (PDTC) is a thiol compound that exerts antioxidant and anti-inflammatory effects. Although the anti-inflammatory function of PDTC in several inflammatory disorders has been reported, the effect of PDTC on *C. acnes*-induced skin inflammation remains unexplored. In the present study, we examined the effect of PDTC on *C. acnes*-induced inflammatory responses and determined the mechanism by using in vitro and in vivo experimental models. We found that PDTC significantly inhibited the expression of *C. acnes*-induced proinflammatory mediators, such as interleukin-1β (IL-1β), interleukin-6 (IL-6), tumor necrosis factor-α (TNF-α), cyclooxygenase-2 (COX-2), inducible nitric oxide synthase (iNOS), and NOD-like receptor (NLR) pyrin domain-containing 3 (NLRP3), in mouse-bone-marrow-derived macrophage (BMDM) cells. PDTC suppressed *C. acnes*-induced activation of nuclear factor-kappa B (NF-κB), which is the major transcription factor for proinflammatory cytokine expression. In addition, we found that PDTC inhibited caspase-1 activation and IL-1β secretion through suppressing NLRP3 and activated the melanoma 2 (AIM2) inflammasome but not the NLR CARD-containing 4 (NLRC4) inflammasome. Moreover, we found that PDTC improved *C. acnes*-induced inflammation by attenuating *C. acnes*-induced IL-1β secretion in a mouse acne model. Therefore, our results suggest that PDTC has potential therapeutic value for the amelioration of *C. acnes*-induced skin inflammation.

## 1. Introduction

Acne vulgaris is a common inflammatory skin disorder caused by excessive sebum production in adolescence, and it can leave scars on the face and persist for one’s lifetime [1]. Acne is caused by various combinations of factors, such as environmental pollution, stress, genetic causes, and bacterial infections [2]. Colonization of *Cutibacterium acnes* (*C. acnes*), a Gram-positive anaerobic bacterium, in sebaceous follicles is known to contribute to the inflammatory responses of acne [3]. *C. acnes* causes macrophages to stimulate the secretion of proinflammatory cytokines, such as IL-1β, IL-6, and TNF-α [4]. Using an in vitro cell culture system, *C. acnes* exposures increased iNOS and COX-2 expression via elevated AP-1/NF-κB activation in RAW264.7, J774A.1, and peritoneal macrophages [5]. In early acne lesions, the IL-17-producing T-cell subset known as Th17 is detected and plays a role in the regulation of adaptive immune responses by secreting cytokines and chemokines that activate effector cells [6,7]. The induction of proinflammatory cytokines by *C. acnes* is mediated by Toll-like receptor 2 (TLR2), a transmembrane protein that plays an important role in the innate immune response to pathogens [8]. TLR2 expression is increased in keratinocytes, monocytes, and macrophages infected with *C. acnes* [9]. When TLR2 is activated by exposure to *C. acnes*, it triggers the activation of the transcription factor NF-κB. NF-κB is bound to IκB, exists in an inactive form in the cytoplasm, and is released as IκBα, which is phosphorylated by stimulation. Phosphorylated IκBα is degraded, and NF-κB migrates to the nucleus to induce the transcription of inflammatory mediators, such as pro-IL-1β, COX-2, and iNOS [10]. NF-κB activation and nuclear translocation in epithelial cells and surrounding cells in inflammatory acne skin tissue has been reported [11,12]. 

The presence of pathogenic microorganisms activates cytosolic multiprotein complex inflammasomes, such as NLRP3, NLRC4, and AIM2, to trigger the inflammatory response and exert antimicrobial host defense mechanisms [13]. NLRP3 and NLRC4 belong to the NLR family of inflammasomes, and the AIM2 inflammasome is a non-NLR family. Inflammasomes oligomerize to recruit apoptosis-associated speck-like protein containing CARD (ASC) and pro-caspase-1, which triggers the self-proteolytic cleavage of pro-caspase-1 into active caspase-1 and subsequent maturation of pro-IL-1β into active IL-1β [13]. 

Antibiotics such as tetracycline, clindamycin, and erythromycin are mainly used to treat acne [14]. However, long-term and improper use of antibiotics is known to cause numerous side-effects, such as skin irritation, redness and hyperpigmentation of the skin, and the development of antibiotic-resistant bacteria [15,16]. Although isotretinoin, benzoyl peroxide (BPO), and synthetic sulfone are efficient methods to treat acne, the degrees of response and tolerance vary among individuals [17,18,19]. Therefore, developing therapeutic drugs with different mechanisms of action for acne treatment is important for broadening the treatment options.

Pyrrolidine dithiocarbamate (PDTC) is a thiol compound that has been shown to exert diverse biological functions. PDTC acts as a radical scavenger, ion chelator, ubiquitin-proteasomal inhibitor, and NF-kB inhibitor [20,21]. The ion chelating property of PDTC has been linked to apoptosis in lung cancer cells, pancreatic adenocarcinoma cells, and rat cortical astrocytes [22,23,24]. The cytoprotective effect of PDTC has been shown in a rat liver injury model by inducing heme oxygenase-1 expression [25,26]. The anti-inflammatory effect of PDTC has been reported in in vivo models of acute and chronic inflammation [25,26,27]. Several reports have shown that the anti-inflammatory effect of PDTC occurs by inhibiting NF-κB activity [28]. For example, PDTC inhibits the transcription of pro-IL-1β, IL-6, IL-8, and colony stimulating factor (GM-CSF) in human endothelial cells via inhibition of NF-κB activation [29]. PDTC inhibits iNOS expression and NO synthesis activity in human hepatic epithelial cells after cytokine stimulation by suppressing NF-κB activation [30]. PDTC also exerts an anti-inflammatory effect by altering the stability of the STAT3-Hsp90 complex in cultured hepatocytes [31]. 

Although the anti-inflammatory effect of PDTC has been reported to occur in several experimental models, the effect of PDTC on *C. acnes*-induced skin inflammation has not been elucidated. In this study, we evaluated the effect of PDTC on *C. acnes*-induced inflammatory signaling pathways using in vitro and in vivo mouse acne models and found that PDTC ameliorates *C. acnes*-induced skin inflammation by inhibiting IL-1β secretion.

## 2. Results

### 2.1. PDTC Suppresses C. acnes-Induced Inflammation in BMDMs

**Figure 1:**
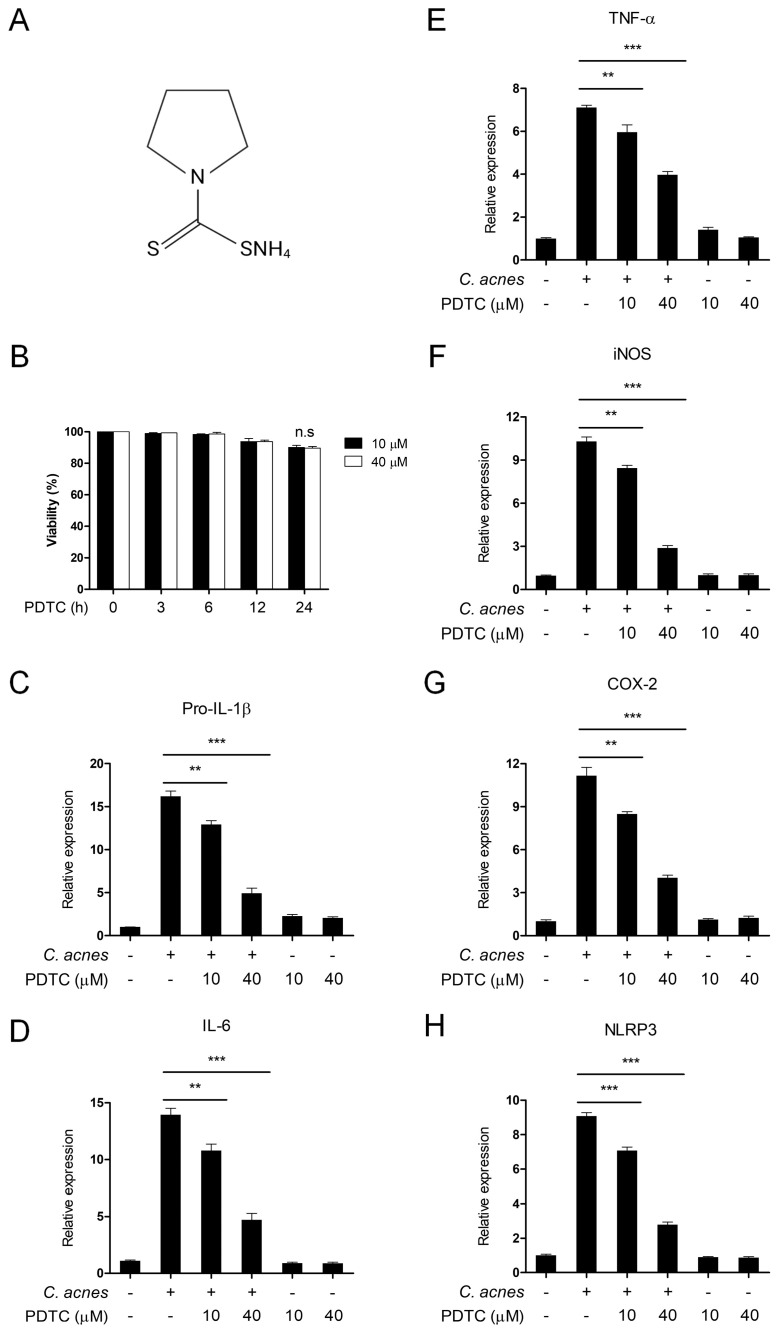
Inhibitory effects of PDTC on the *C. acnes*-induced inflammatory response. (**A**) Chemical structure of PDTC. (**B**) Mouse BMDMs were treated with 10 μM PDTC and 40 μM PDTC for the indicated times. The viability of cells was assessed by MTT assay. (**C**–**H**) Mouse BMDMs were pretreated with PDTC (10 μM and 40 μM) for 30 min and then incubated with heat-killed *C. acnes* (1 × 10^6^ CFU/mL) for 6 h. The mRNA (pro-IL-1β, IL-6, TNF-α, iNOS, COX-2, and NLRP3) levels were measured using quantitative real-time PCR. The results are represented as the means ± SDs of three independent experiments. n.s, nonsignificant. ** *p* < 0.01. *** *p* < 0.001.

To elucidate the possible effects of PDTC on *C. acnes*-induced inflammatory signaling pathways, we first determined the noncytotoxic concentration of PDTC in BMDMs. The structure of PDTC is shown in Figure 1A. BMDMs were treated with 10 and 40 μM PDTC for up to 24 h, and the viability of cells was measured by MTT assay. PDTC did not induce any significant cytotoxicity in BMDMs until 24 h had passed (Figure 1B). Based on these results, we used 10 and 40 μM PDTC for all of the following experiments. We next examined the effect of PDTC on the mRNA expression of *C. acnes*-induced inflammatory mediators using quantitative real-time PCR (qPCR) analysis (Figure 1C–H). As shown in Figure 1C–H, pretreatment with PDTC caused a dose-dependent inhibition of the mRNA levels of various *C. acnes*-induced inflammatory mediators, such as pro-IL-1β, IL-6, TNF-α, iNOS, COX-2, and NLRP3. These results indicate that PDTC has an inhibitory effect on *C. acnes*-induced inflammatory signaling pathways.

### 2.2. PDTC Inhibits the Protein Expression of C. acnes-Induced Inflammatory Mediators

**Figure 2:**
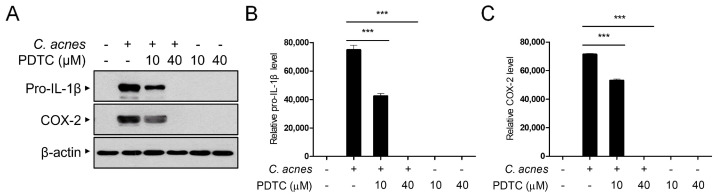
Inhibitory effects of PDTC on the expression of *C. acnes*-induced inflammatory proteins. Mouse BMDMs were pretreated with PDTC (10 and 40 μM) for 30 min and then incubated with heat-killed *C. acnes* (1 × 10^6^ CFU/mL) for 6 h. Pro-IL-1β, COX-2, and β-actin protein levels were detected by Western blot analysis (**A**), and then the relative levels of protein bands were quantified (**B**,**C**). The results are represented as the means ± SDs of three independent experiments. *** *p* < 0.001.

We next examined the effect of PDTC on the protein expression of *C. acnes*-induced proinflammatory mediators, such as pro-IL-1β and COX-2, using Western blot analysis. As shown in Figure 2A,B, pretreatment with PDTC suppressed *C. acnes*-induced pro-IL-1β protein expression in a dose-dependent manner. The level of COX-2 protein expression was consistently suppressed by PDTC treatment, confirming that PDTC exerts an anti-inflammatory effect on *C. acnes*-triggered inflammatory signaling pathways (Figure 2A,C).

### 2.3. PDTC Inhibits C. acnes-Induced NF-κB Activation

**Figure 3:**
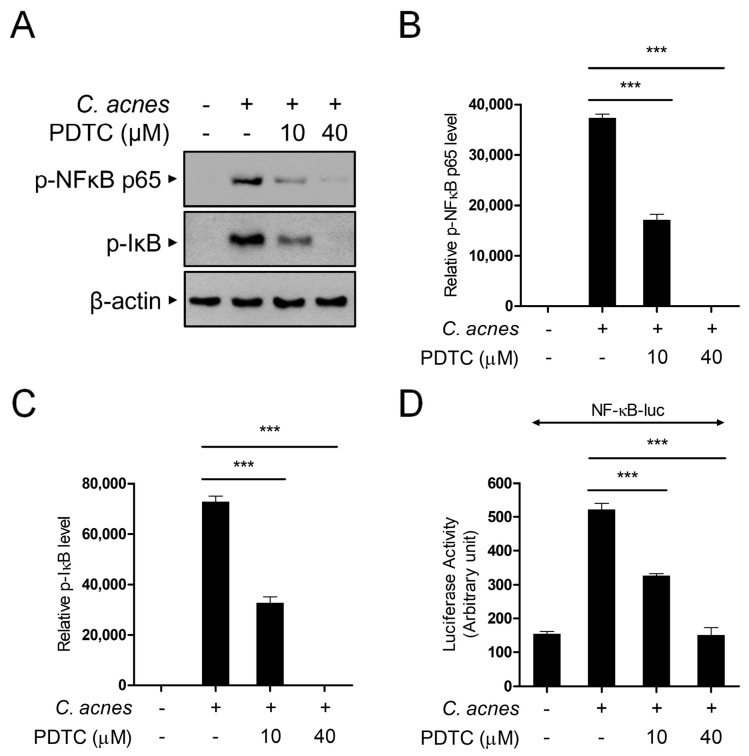
PDTC suppresses *C. acnes*-induced NF-κB transcriptional activation. (**A**–**C**) Mouse BMDMs were pretreated with PDTC (10 μM and 40 μM) for 30 min and then incubated with heat-killed *C. acnes* (1 × 10^6^ CFU/mL) for 30 min. p-NF-κB (p65), p-IκB, and β-actin protein levels were detected by Western blot analysis (**A**), and then the relative levels of protein bands were quantified (**B**,**C**). (**D**) RAW264.7 cells were transfected with the NF-κB-luciferase reporter. After 24 h, the cells were pretreated with PDTC (10 μM and 40 μM) for 30 min and then incubated with heat-killed *C. acnes* (1 × 10^6^ CFU/mL) for 6 h. The results are represented as the means ± SDs of three independent experiments. *** *p* < 0.001.

We next determined the signaling cascades involved in the PDTC-mediated anti-inflammatory effect in response to *C. acnes*. As PDTC is widely used as an inhibitor of NF-κB activation, we examined whether PDTC-mediated suppression of *C. acnes*-induced inflammatory signaling occurs through the inhibition of NF-κB activation. We measured the phosphorylation of NF-κB (p65) by Western blot analysis (Figure 3A,B). Treatment of BMDMs with *C. acnes* induced the phosphorylation of NF-κB, and the level of phosphorylation was significantly suppressed by PDTC in a dose-dependent manner (Figure 3A,B). In accordance with this result, the phosphorylation of IκB, which causes NF-κB to migrate to the nucleus, was also inhibited by PDTC treatment (Figure 3A,C). To further confirm the inhibitory effect of PDTC on *C. acnes*-induced NF-κB activation, we measured *C. acnes*-induced NF-κB transcriptional activation using reporter analysis (Figure 3D). As shown in Figure 3D, PDTC consistently suppressed NF-κB-dependent gene transcriptional activation, indicating that the inhibitory effect of PDTC on *C. acnes*-induced inflammatory gene transcription is dependent on NF-κB signaling.

### 2.4. PDTC Inhibits C. acnes-Triggered NLRP3 and AIM2 Inflammasome Activation

**Figure 4:**
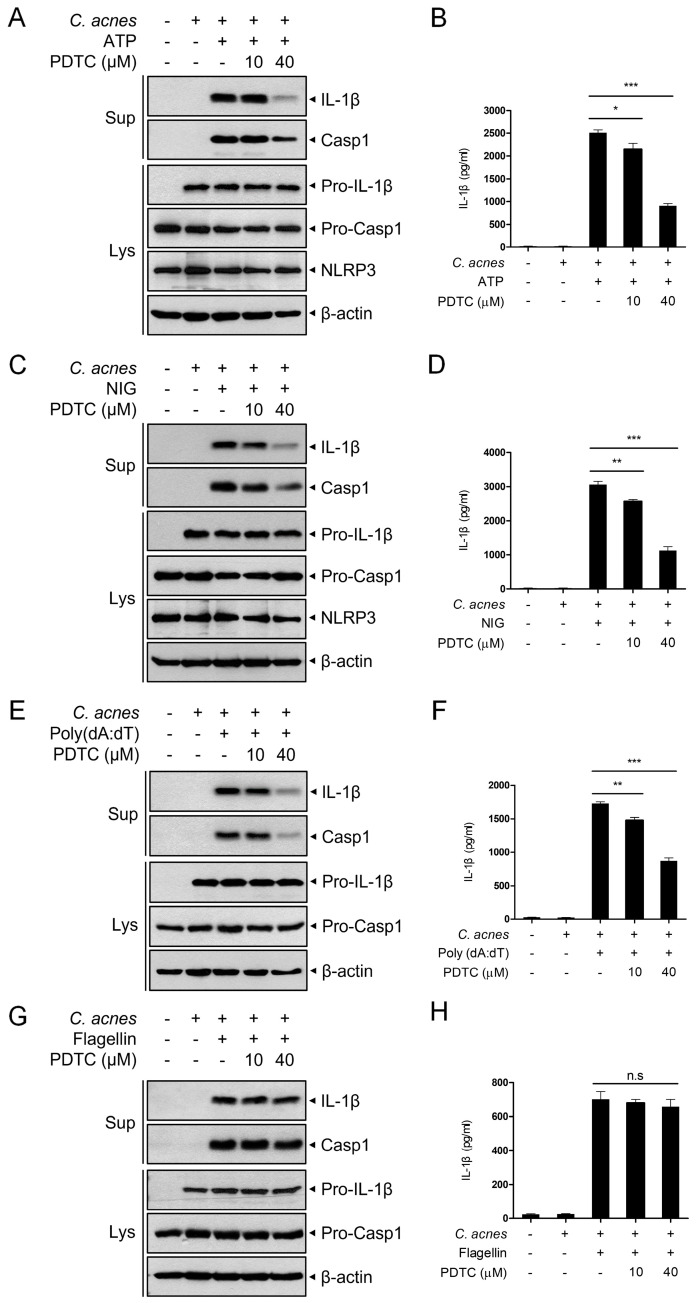
Inhibitory effects of PDTC on *C. acnes*-induced inflammasome activation. (**A**–**D**) Mouse BMDMs were primed with heat-killed *C. acnes* (1 × 10^6^ CFU/mL) for 3 h and then treated with PDTC (10 μM and 40 μM) for 30 min before either ATP (5 mM) or nigericin (10 μM) incubation for 1 h. Culture supernatants (Sup) and cell lysates (Lys) were immunoblotted with anti-IL-1β, anti-caspase-1 and anti-NLRP3 antibodies (**A**,**D**). The secreted IL-1β level was measured using ELISA (**B**,**C**). (**E**–**H**) Mouse BMDMs were primed with heat-killed *C. acnes* (1 × 10^6^ CFU/mL) for 3 h and then treated with PDTC (10 μM and 40 μM) for 30 min before poly (dA:dT) (10 μg/mL) transfection with lipofectamine 3000 or flagellin treatment (5 μg/mL) for 4 h. Culture supernatants (Sup) and cell lyastes (Lys) were immunoblotted with the indicated antibodies (**E**,**G**). The secreted IL-1β level was measured using ELISA (**F**,**H**). The results are represented as the means ± SDs of three independent experiments. * *p* < 0.05, ** *p* < 0.01, *** *p* < 0.001; n.s, nonsignificant.

The secretion of active IL-1β was promoted by inflammasome pathways. We next investigated whether the inflammasome complex is involved in the PDTC-mediated suppression of the inflammatory signaling cascade in response to *C. acnes*. We first analyzed NLRP3 inflammasome activation in response to *C. acnes*. Mouse BMDMs were primed with *C. acnes* and then treated with ATP, which is known to activate the NLRP3 inflammasome, in the presence or absence of PDTC (Figure 4A,B). The application of ATP induced the secretion of active IL-1β and cleaved caspase-1 in the supernatant, and PDTC inhibited this secretion (Figure 4A). The decreased secretion of active IL-1β protein was further confirmed using ELISA (Figure 4B). In accordance with these results, PDTC suppressed IL-1β and caspase-1 secretion induced by the alternative NLRP3 inflammasome activator nigericin (Figure 4C,D). To investigate the effect of PDTC on other inflammasomes, *C. acnes*-primed BMDMs were treated with the AIM2 inflammasome activator poly (dA:dT). PDTC inhibited poly (dA:dT)-induced IL-1β and caspase-1 secretion (Figure 4E,F). However, PDTC did not alter active IL-1β and caspase-1 secretion levels in flagellin-treated BMDMs (flagellin being an NLRC4 inflammasome activator) (Figure 4G,H). These results suggest that PDTC inhibits the NLRP3 and AIM2 inflammasomes but not the NLRC4 inflammasome.

### 2.5. PDTC Inhibits C. acnes-Induced Skin Inflammation In Vivo

**Figure 5:**
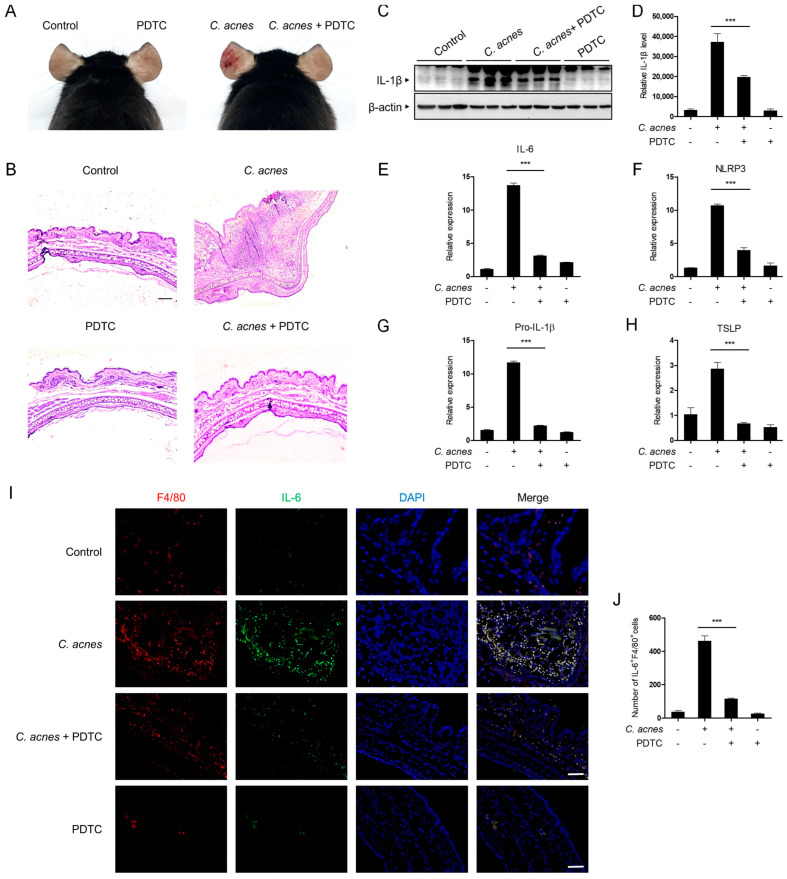
Inhibitory effects of PDTC in a mouse acne model. (**A**) Live *C. acnes* (1 × 10^8^ CFU per 20 μL in PBS) were injected into mouse ears with or without PDTC (100 mg/kg). At 24 h after injection, mouse ears were photographed. (**B**) Ear tissues were sectioned and stained with H&E. Scale Bars: 100 μm. (**C**) IL-1β and β-actin protein levels were detected in ear tissues using Western blot analysis. (**D**) The relative levels of protein bands were quantified. (**E**–**H**) The mRNA (IL-6, pro-IL-1β, NLRP3, and TSLP) levels were measured in mouse ear tissues using quantitative real-time PCR. (**I**) Immunofluorescence tissue images were obtained for F4/80 (red) and IL-6 (green) in acne lesions. DAPI (blue) was used to detect nuclei. The images were captured separately and merged. Scale bars: 50 μm. (**J**) The number of IL-6^+^F4/80^+^cells in the image was counted. The results are represented as the means ± SDs of three independent experiments. *** *p* < 0.001.

**Figure 6:**
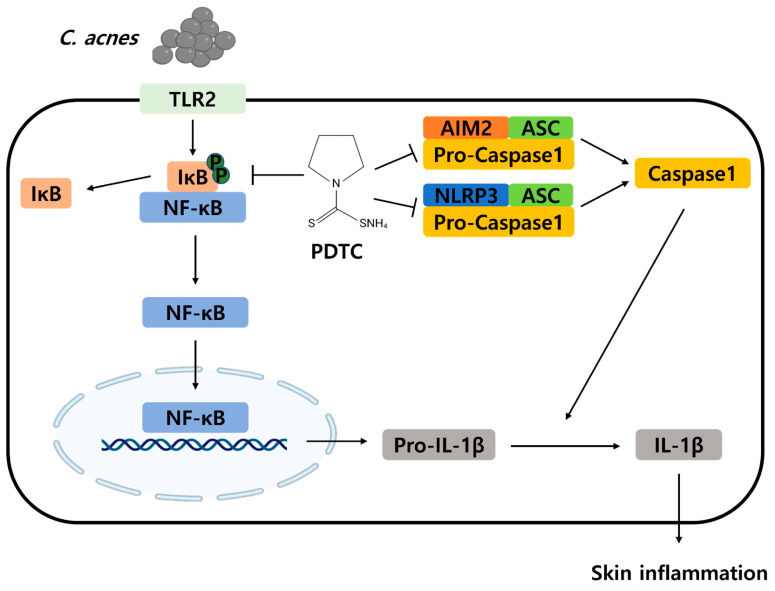
A schematic model of PDTC in the inhibition of *C. acnes*-induced skin inflammation. PDTC inhibits the secretion of IL-1β by inhibiting *C. acnes*-triggered NF-κB activation and inflammasome activation.

We next examined the pathophysiological effect of PDTC using a mouse acne model. For this experiment, live *C. acnes* were injected into mouse ears with or without PDTC. At 24 h after *C. acnes* injection, histological changes were monitored (Figure 5A). The *C. acnes*-injected ear showed cutaneous erythema, a typical symptom of inflammation, but the PDTC-treated ear exhibited markedly reduced erythema (Figure 5A). To further investigate the inflammatory reactions, pathophysiological changes in the ear tissues were monitored with hematoxylin and eosin (H&E) staining (Figure 5B). Inoculation with *C. acnes* induced swelling and an increase in the number of inflammatory cells that infiltrated into the dermis, and PDTC attenuated these reactions (Figure 5B). To evaluate the inhibitory effects of PDTC on *C. acnes*-induced ear inflammation, we examined IL-1β protein expression in the ear using Western blot analysis (Figure 5C,D). The ear IL-1β protein expression levels were significantly increased by *C. acnes* injection, and PDTC treatment suppressed this increase in IL-1β expression (Figure 5C,D). To further evaluate the inhibitory effect of PDTC on *C. acnes*-induced ear inflammation, we measured the mRNA levels of inflammatory markers in ear tissue using qPCR analysis. IL-6, IL-1β, and NLRP3 mRNA levels were upregulated in *C. acnes*-treated ear tissues, and PDTC application decreased their production (Figure 5E–G). Consistently, PDTC lowered the mRNA level of TSLP, a cytokine that is known to be upregulated in skin inflammation (Figure 5H). We next examined whether tissue macrophages in acne lesions are involved in the PDTC-mediated anti-inflammatory effects using immunofluorescence analysis (Figure 5I,J). A higher prevalence of F4/80^+^ macrophages, which is a well-established murine macrophage marker, in *C. acnes*-treated ear tissues than in control ear tissues was observed, and PDTC treatment reduced the number of F4/80^+^ macrophages (Figure 5I,J). Furthermore, the number of IL-6-expressing F4/80^+^ macrophages was consistently lower in PDTC-treated acne lesions (Figure 5I,J). Collectively, these results suggest that PDTC ameliorates *C. acnes*-induced skin inflammation in vivo (Figure 6).

## 3. Discussion

Acne is a multifactorial skin disease that affects a large population of adolescents. The complex etiology of acne includes increased sebum production, damaged hair-follicle keratinization, and colonization of sebaceous glands by *C. acnes* to induce inflammation. Although the presence of *C. acnes* may not be a prerequisite factor for the initiation of inflammation, it may exacerbate or intensify acne symptoms in humans [32,33].

Topical and systemic treatments have been included in the current treatments for acne. Retinoids, such as tretinoin and erythromycin, are commonly used for their topical anti-inflammatory effects [34]. Salicylic acid and benzoyl peroxide have been used as antibacterial agents to treat mild-to-moderate acne [35]. Tetracycline family antibiotics, such as minocycline and doxycycline, are oral antibiotics that are commonly prescribed to treat moderate acne [35]. For patients with severe acne, oral isotretinoin is used to control inflammation [36]. In addition, a low dose of estrogen can be prescribed for anti-androgenic effects [34]. Nonetheless, these treatments can cause irritation of the skin and low patient adherence and compliance [37]. Although isotretinoin is generally regarded as safe, embryotoxic and teratogenic cases have been reported [37]. Furthermore, long-term use of antibiotics causes the generation of antibiotic-resistant acne bacteria [38]. Therefore, it is necessary to continuously develop new therapies that are safe and effective and have few side-effects.

PDTC belongs to the dithocarbamate (DTC) class and has been used in medicine, agriculture, and industry for more than 20 years. The potential clinical use of the DTC class has been reported for the treatment of various diseases, such as ocular inflammation, rhinovirus infections, and *Staphylococcus aureus* infection [39,40,41]. The administration of DTC did not induce any major adverse biological reactions and shows potential to reduce the incidence of opportunistic infections in patients with symptomatic HIV infection [42]. Similarly to other DTC classes, PDTC has been examined for its preclinical safety and potential use in the intranasal treatment of human rhinovirus infections using in vitro and in vivo models [43]. PDTC has been shown to act as an antioxidant and radical scavenger and influences biological processes such as apoptosis, enzyme inhibition, modulation of transcription, and inhibition of inflammation [20,21]. The anti-inflammatory effect of PDTC is mostly associated with the inhibition of NF-κB activity [27,29]. In accordance with these reports, we observed that PDTC suppressed *C. acnes*-induced expression of proinflammatory cytokines by inhibiting NF-κB transcriptional activation in BMDMs. 

To produce active proinflammatory cytokines, such as IL-1β and IL-18, caspase-1 must be activated by high-molecular-weight platforms called inflammasomes [44,45]. NLRP3, NLRC4, and AIM2 are the most well-characterized inflammasomes and have been reported to be activated by a wide range of pathogenic signals that are derived from microbial- and host-derived triggers [44,46]. It has been reported that *C. acnes* can trigger an innate immune response in mouse skin and that inflammation is dependent on IL-1β and the NLRP3 inflammasome of myeloid cells [47]. In skin biopsies of inflammatory acne lesions, macrophages contained phagocytized *C. acnes* and expressed the NLRP3 inflammasome [48]. In accordance with these reports, we observed that *C. acnes* triggers the activation of NLRP3, NLRC4, and AIM2 inflammasomes in BMDMs; and PDTC treatment inhibited NLRP3 and AIM2 inflammasome activation. However, PDTC did not suppress NLRC4 inflammasome activation, indicating that PDTC was not involved in inhibiting the activation of flagellin-mediated inflammasomes.

Based on the in vitro results, we investigated the in vivo effect of PDTC using a mouse acne model. Intradermal injection of *C. acnes* into the mouse ear induced edema and redness, typical symptoms of skin inflammation, and PDTC treatment alleviated these skin pathologies. PDTC treatment consistently lowered the expression of *C. acnes*-induced inflammatory mediators, such as NLRP3, IL-1β, IL-6, and TSLP, in mouse ears, confirming the in vivo effect of PDTC. As PDTC decreased the secretion of proinflammatory cytokines and chemokines in macrophages in inflammatory acne lesions, we speculate that PDTC might lower the Th17 responses, which are involved in the pathogenesis of acne.

## 4. Materials and Methods

### 4.1. Materials

Pyrrolidine dithiocarbamate (PDTC) and ATP were purchased from Sigma-Aldrich (St. Louis, MO, USA). Nigericin was purchased from Tocris (Bristol, UK). *Salmonella typhimurium* flagellin and poly (dA:dT) were purchased from InvivoGen (San Diego, CA, USA). Anti-phospho-IκB, anti-phospho-NF-κB (p65), anti-IL-6, and anti-COX-2 antibodies were purchased from Cell Signaling Technology (Danvers, MA, USA). The anti-IL-1β antibody was purchased from R&D Systems (Minneapolis, MN, USA). Anti-NLRP3 and anti-caspase-1 antibodies were purchased from AdipoGen Life Science (San Diego, CA, USA). The anti-β-actin antibody was purchased from Santa Cruz Biotechnology (Dallas, TX, USA). The anti-mouse F4/80 antibody was purchased from Biolegend (San Diego, CA, USA). 

### 4.2. C. acnes

*C. acnes* (KCTC3314) were obtained from the Korean Culture Center of Microorganisms (Seoul, Republic of Korea). *C. acnes* were cultured in reinforced clostridial medium (Merck Millipore, Darmstadt, Germany) using anaerobic Gas-Pak at 37 °C. *C. acnes* were centrifuged at 4500 rpm for 20 min at 4 °C, and the bacterial pellets were washed with PBS before use.

### 4.3. Cell Culture

Bone-marrow-derived macrophages (BMDMs) were prepared as described previously [49]. Bone marrow progenitor cells were isolated from C57BL/6 mice (8–12 weeks old) and differentiated into BMDMs in the presence of 30% L929 cell-conditioned medium (LCM). The BMDMs were cultured in DMEM containing 30% LCM, 10% heat-inactivated fetal bovine serum (FBS), penicillin, and streptomycin (Invitrogen, Carlsbad, CA, USA). RAW264.7 cells were purchased from American Type Culture Collection (Rockville, MD, USA) and cultured in DMEM containing 10% heat-inactivated fetal bovine serum FBS, penicillin, and streptomycin. All cells were maintained at 37 °C in a 5% CO_2_ incubator.

### 4.4. MTT Assay

BMDMs (1 × 10^6^ cells/mL) were plated in 12-well plates. After 24 h, the BMDMs were treated with PDTC at the indicated times or concentrations and incubated with MTT (1 mg/mL) (Sigma-Aldrich, St. Louis, MO, USA) at 37 °C for 1 h. MTT formazan was dissolved in dimethyl sulfoxide (DMSO), and the absorbance was measured at 570 nm.

### 4.5. Reporter Gene Assay Analysis

RAW264.7 cells were transfected with the NF-κB-Luc reporter and control *Renilla*-Luc reporter using Lipofectamine 3000 (Invitrogen, Carlsbad, CA, USA). Luciferase activity was measured using the Dual-Luciferase Assays System (Promega, Madison, WI, USA).

### 4.6. ELISA

The culture supernatant was collected into a tube, and the concentrations of IL-1β were measured in accordance with the manufacturer’s protocol (R&D Systems, Minneapolis, MN, USA).

### 4.7. Quantitative Real-Time PCR

Total RNA was isolated using TRIzol reagent (Invitrogen, Carlsbad, CA, USA), and cDNA synthesis was performed with M-MLV reverse transcriptase (Promega, Madison, WI, USA). The product was amplified with SYBR Green real-time PCR Master Mix (TOYOBO, Osaka, Japan) using the following primers: pro-IL-1β, 5′-GCCACCTTTTGACAGTGATGAG-3′ (forward) and 5′-AGTGATACTGCCTGCCTGAAG-3′ (reverse); IL-6, 5′-TACCACTTCACAAGTCGGAGGC-3′ (forward) and 5′-CTGCAAGTGCATCATCGTTGTTC-3′ (reverse); TNF-α, 5′-CCCTCACACTCACAAACCAC-3′ (forward) and 5′-ACAAGGTACAACCCATCGGC-3′ (reverse); iNOS, 5′-GCTGGTTTGAAACTTCTCAG-3′ (forward) and 5′-GAAGGGTGTCGTGAAAAATC-3′ (reverse); COX-2, 5′-TTGGAGGCGAAGTGGGTTTT-3′ (forward) and 5′-TGGGAGGCACTTGCATTGAT-3′ (reverse); NRLP3, 5′-GACCGTGAGGAAAGGACCAG-3′ (forward) and 5′- GGCCAAAGAGGAATCGGACA-3′ (reverse); TSLP, 5′-CCCTTCACTCCCCGACAAAA-3’ (forward) and 5’-GCAGTGGTCATTGAGGGCTT-3’ (reverse); and β-actin, 5′-AGAGGGAAATCGTGCGTGAC-3′ (forward) and 5′-CGATAGTGATGACCTGACCGT-3′ (reverse). All samples were run in triplicate, and the mRNA level was normalized to β-actin. The values were analyzed using AriaMX (Agilent, Santa Clara, CA, USA).

### 4.8. Western Blot Analysis

BMDMs (1 × 10^6^ cells/mL) were plated in 12-well plates. After 24 h, the BMDMs were primed with heat-killed *C. acnes* (1 × 10^6^ CFU/mL) for 6 h and then treated with PDTC (10 and 40 μM). For the analysis of NLRP3 inflammasome activation, cells were treated with either ATP (5 mM) or nigericin (10 μM) for 1 h. For AIM2 inflammasome activation, the poly (dA:dT) (10 μg/mL) was transfected with Lipofectamine 3000 according to the manufacturer’s instructions for 6 h. For the NLRC4 inflammasome activation, the cells were treated with flagellin (5 μg/mL) for 4 h. The culture supernatant was collected in the tube and centrifuged at 2000 rpm for 5 min to remove detached cells. The cells were resuspended in lysis buffer containing 50 mM Tris-Cl (pH 8.0), 1% Nonidet P-40, 150 mM NaCl, 1 mM EGTA, 10% glycerol, 10 mM NaF, protease inhibitor, 0.2 mM phenylmethylsulfonylfluoride (PMSF), and 1 mM Na_3_VO_4_ and incubated on ice for 30 min. After centrifugation, the lysate (Lys) was collected into a tube, and the proteins were separated by SDS-PAGE and transferred to a nitrocellulose membrane. The membranes were blocked in TBST buffer containing 5% skim milk. The blocked membranes were incubated with the primary antibody overnight at 4 °C and subsequently incubated with the corresponding secondary antibody for 1 h. The bands were then visualized with enhanced chemiluminescence solution.

### 4.9. In Vivo Mouse Acne Model

C57BL/6 mice were purchased from Orient Bio Inc. (Seongnam, Republic of Korea) and bred at the Animal Laboratory Center of Kangwon National University. All experiments were approved by the Institutional Animal Care and Use Committee (IACUC, KW-201026-1, Kangwon National University, Republic of Korea). Mice (three per group) were injected with live *C. acnes* (1 × 10^8^ CFU per 20 μL in PBS) into the ears with or without PDTC (100 mg/kg). After 24 h, mice were sacrificed, and ear tissues were then collected for further analysis. For the immunofluorescence analysis, tissue sections were deparaffinized and blocked with 5% bovine serum albumin (BSA) for 30 min. Sections were then incubated with the phycoerythrin (PE)-conjugated anti-mouse F4/80 antibody (1:400) and anti-IL-6 antibody (1:500) overnight. After incubation with FITC-conjugated anti-rabbit IgG antibody (1:400) and 40,6-diamidino-2-phenylindole (DAPI) for 1 h, tissue sections were mounted in mounting medium (Abcam, Cambridge, MA, USA), and the image was analyzed using fluorescence microscopy.

### 4.10. Statistics

Densitometric scans of the Western blot analyses were quantified using ImageJ software (NIH, Bethesda, MD, USA). GraphPad Prism (GraphPad Software, Inc., San Diego, CA, USA) was used for the data analyses, and the results are presented as the means ± SDs. Differences between the experimental group and the control group were analyzed using Student’s *t*-tests. Comparisons between multiple groups were analyzed using ANOVA, followed by Bonferroni post hoc testing. A value of *p* < 0.05 was considered statistically significant. * *p* < 0.05; ** *p* < 0.01; *** *p* < 0.001.

## 5. Conclusions

In the present study, we demonstrated that PDTC attenuates *C. acnes*-induced inflammatory signaling by inhibiting NF-κB and inflammasome activation in BMDMs and in acne-model mice. Our results show for the first time the regulation of *C. acnes*-induced skin inflammation by PDTC and suggest that PDTC could be a potential alternative agent for the clinical treatment of acne. Due to its safety, lack of side-effects, and low cost, PDTC might be comparable to the well-known standard medicines for improving acne symptoms.

## Data Availability

Not applicable.
